# Supplementary material for: Optimization of differentiation and transcriptomic profile of THP-1 cells into macrophage by PMA
Source: PLoS One. 2023 Jul 17;18(7):e0286056. doi: 10.1371/journal.pone.0286056 (PMC10351730; doi:10.1371/journal.pone.0286056)
Supplement: S1 File — (ZIP) [file pone.0286056.s001.zip › supporting files/RNA-seq report.zip!/RNA-seq report/4_GOEnrichment_result/induce_vs_control/DAG/induce_vs_control.DEG_down_bp_DAG.pdf]

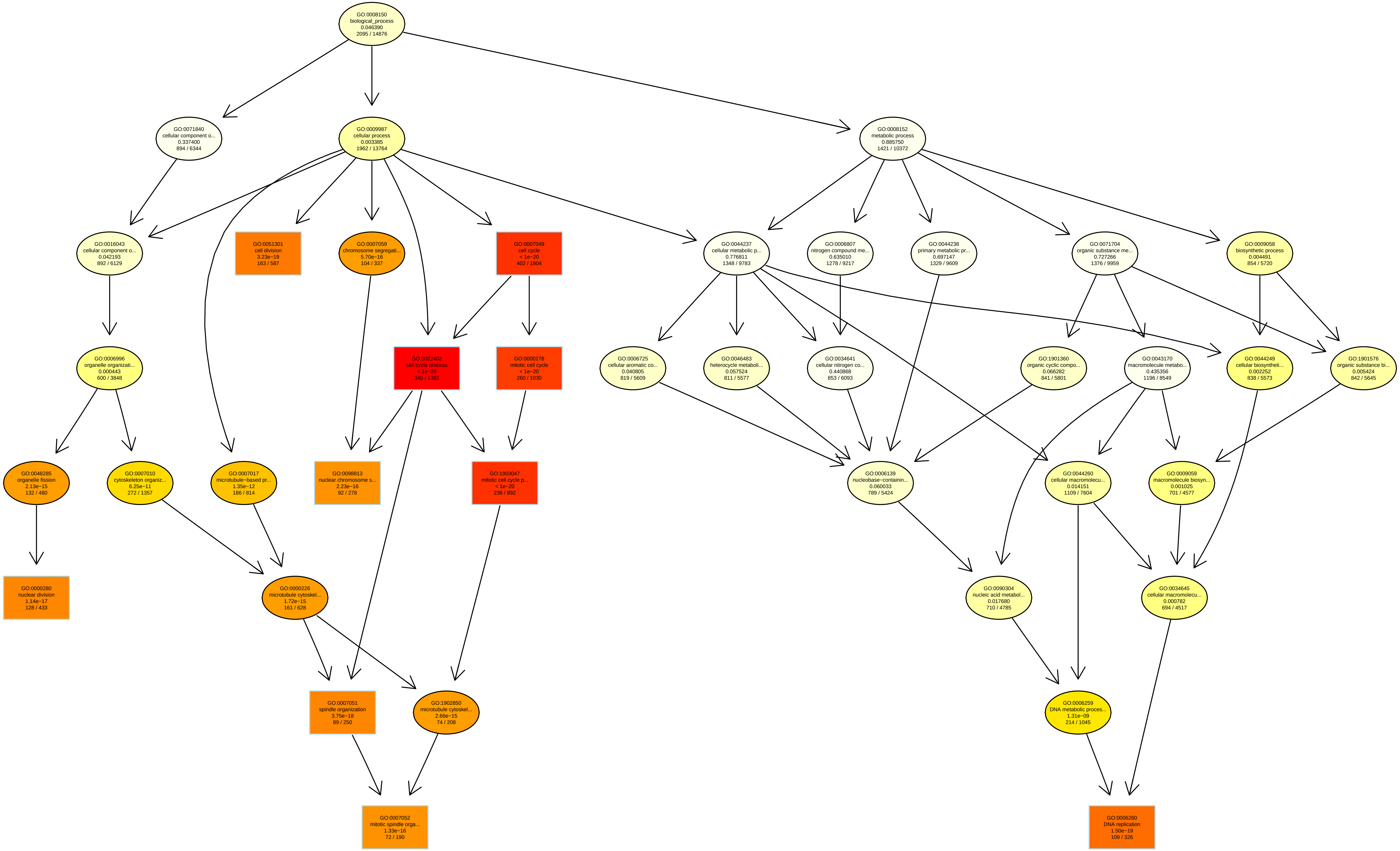
GO:0008150
biological_process
0.046390
2095 / 14876
GO:0071840
cellular component o...
0.337400
894 / 6344
GO:0009987
cellular process
0.003385
1962 / 13764
GO:0008152
metabolic process
0.885750
1421 / 10372
GO:0016043
cellular component o...
0.042193
892 / 6129
GO:0051301
cell division
3.23e−19
163 / 587
GO:0007059
chromosome segregati...
5.70e−16
104 / 337
GO:0007049
cell cycle
< 1e−20
402 / 1804
GO:0044237
cellular metabolic p...
0.776811
1348 / 9783
GO:0006807
nitrogen compound me...
0.635010
1278 / 9217
GO:0044238
primary metabolic pr...
0.697147
1329 / 9609
GO:0071704
organic substance me...
0.727266
1376 / 9959
GO:0009058
biosynthetic process
0.004491
854 / 5720
GO:0006996
organelle organizati...
0.000443
600 / 3848
GO:0022402
cell cycle process
< 1e−20
340 / 1382
GO:0000278
mitotic cell cycle
< 1e−20
260 / 1030
GO:0006725
cellular aromatic co...
0.040805
819 / 5609
GO:0046483
heterocycle metaboli...
0.057524
811 / 5577
GO:0034641
cellular nitrogen co...
0.440868
853 / 6093
GO:1901360
organic cyclic compo...
0.066282
841 / 5801
GO:0043170
macromolecule metabo...
0.435356
1196 / 8549
GO:0044249
cellular biosyntheti...
0.002252
838 / 5573
GO:1901576
organic substance bi...
0.005424
842 / 5645
GO:0048285
organelle fission
2.13e−15
132 / 480
GO:0007010
cytoskeleton organiz...
6.25e−11
272 / 1357
GO:0007017
microtubule−based pr...
1.35e−12
186 / 814
GO:0098813
nuclear chromosome s...
2.23e−16
92 / 278
GO:1903047
mitotic cell cycle p...
< 1e−20
236 / 892
GO:0006139
nucleobase−containin...
0.060033
789 / 5424
GO:0044260
cellular macromolecu...
0.014151
1109 / 7604
GO:0009059
macromolecule biosyn...
0.001025
701 / 4577
GO:0000280
nuclear division
1.14e−17
128 / 433
GO:0000226
microtubule cytoskel...
1.72e−15
161 / 628
GO:0090304
nucleic acid metabol...
0.017680
710 / 4785
GO:0034645
cellular macromolecu...
0.000782
694 / 4517
GO:0007051
spindle organization
3.75e−18
89 / 250
GO:1902850
microtubule cytoskel...
2.66e−15
74 / 208
GO:0006259
DNA metabolic proces...
1.31e−09
214 / 1045
GO:0007052
mitotic spindle orga...
1.33e−16
72 / 190
GO:0006260
DNA replication
1.50e−19
109 / 326
